# Immunometabolism and male reproductive function: linking inflammation, oxidative stress, and declining fertility

**DOI:** 10.3389/fimmu.2025.1736492

**Published:** 2025-12-09

**Authors:** Jiedong Zhou, Shian Hu, Yong Ouyang, Yucheng Kong, Min Liu

**Affiliations:** 1 The First Affiliated Hospital of Gannan Medical University, Ganzhou, Jiangxi, China; 2 Gannan Medical University, Ganzhou, Jiangxi, China; 3 Department of Urology, the First Affiliated Hospital of Gannan Medical University, Ganzhou, Jiangxi, China

**Keywords:** male infertility, immunometabolism, testicular immunity, oxidative stress, gut microbiota, metabolic signaling

## Abstract

**Background:**

Male infertility accounts for approximately 50% of all infertility cases, and its pathogenesis is highly complex. Beyond traditional factors such as genetics, endocrine disorders, and infections, growing evidence indicates that dysregulation of immunometabolism plays a pivotal role in the onset and progression of male reproductive dysfunction.

**Objective:**

This review aims to systematically elucidate the role of immunometabolism in male reproductive health, focusing on the complex interplay among inflammation, oxidative stress, and metabolic imbalance. Additionally, it seeks to summarize potential therapeutic targets and outline future research directions.

**Methods:**

A narrative review was conducted in accordance with the SANRA (Scale for the Assessment of Narrative Review Articles) guidelines. Relevant studies published between January 2010 to March 2025 were retrieved from PubMed, Embase, and Web of Science using keywords such as “immunometabolism,” “testis,” “male infertility,” and “oxidative stress.”

**Results:**

Testicular immune homeostasis depends on the metabolic coordination among Sertoli cells, Leydig cells, and local immune cells. Aberrant immunometabolism disrupts the blood–testis barrier and endocrine balance by enhancing glycolysis, suppressing oxidative phosphorylation, and promoting the accumulation of reactive oxygen species (ROS), thereby impairing spermatogenesis and testosterone synthesis. Systemic metabolic inflammation induced by obesity, diabetes, and gut microbiota dysbiosis further exacerbates testicular dysfunction through the mTOR/HIF-1α signaling axis and the “gut–immune–gonadal axis.” Pharmacological modulation of key immunometabolic regulators, including AMPK, SIRT1, and PPARγ, has been shown to improve sperm quality and hormone levels in experimental models.

**Conclusion:**

Immunometabolism serves as a crucial mechanistic bridge linking inflammation, oxidative stress, and the decline of male fertility. Future studies integrating multi-omics and spatial analysis technologies are expected to delineate immunometabolic phenotypes associated with male infertility, paving the way for precision diagnosis and personalized therapeutic interventions.

## 1 Introduction

Male infertility accounts for approximately 50% of all infertility cases, and its pathogenesis is highly complex, being influenced by multiple factors including genetics, endocrine regulation, infections, environmental exposure, and immune dysfunction (1). In recent years, research has progressively shifted beyond the traditional focus on spermatogenic failure, hormonal abnormalities, and reproductive tract infections toward recognizing the interplay between the immune and metabolic systems—referred to as immunometabolism—as a key determinant of male reproductive health (2).

As an emerging interdisciplinary field, immunometabolism highlights the intimate connection between immune cell function and metabolic state, offering new insights into immune regulation and holding promise for the development of novel therapeutic strategies (3, 4). Metabolic pathways not only provide energy and biosynthetic precursors for immune cells but also directly dictate their activation, differentiation, and effector functions (5). Metabolic imbalances can drive the pro-inflammatory polarization of immune cells, leading to chronic inflammation and subsequent dysfunction of multiple organ systems, including the reproductive system (6).

Within the male reproductive system, the testis exhibits a unique immune privilege that prevents autoimmune responses against sperm antigens. The maintenance of testicular immune tolerance depends on the precise coordination of energy metabolism, redox balance, and lipid homeostasis. These interconnected metabolic networks shape immune cell phenotypes and are fundamental to sustaining reproductive immune stability (7) evidence suggests that disruption of these immunometabolic pathways impairs the testicular microenvironment, resulting in defective spermatogenesis, hormonal dysregulation, and compromised fertility (8).

This review systematically summarizes the mechanistic roles of immunometabolism in male reproductive health, focusing on the interplay between inflammation, oxidative stress, and metabolic imbalance. It further discusses potential therapeutic targets and future research directions.

## 2 Methods literature search strategy

**Figure 1:**
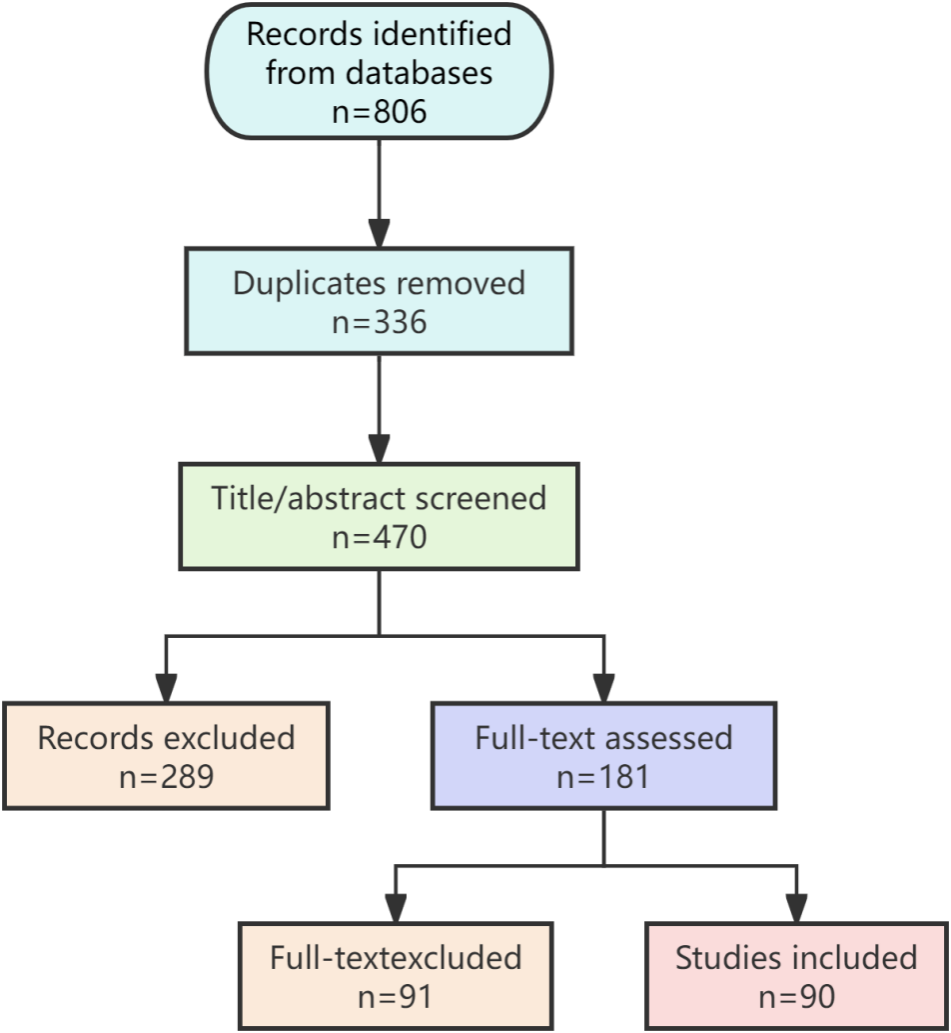
Flow diagram of the study selection process.

This narrative review was conducted in accordance with the Scale for the Assessment of Narrative Review Articles (SANRA) guidelines to ensure methodological rigor. A systematic literature search identified 806 records. After a multi-stage screening process, 90 studies were included for qualitative synthesis (**Figure 1**).

A systematic literature search was performed to identify relevant publications spanning from January 2010 to March 2025. The primary databases interrogated were PubMed, Web of Science, and Embase. The search strategy employed a combination of the following keywords and MeSH terms: (“immunometabolism” OR “immune metabolism”) AND (“testis” OR “testicular” OR “male infertility” OR “spermatogenesis” OR “semen quality”) AND (“oxidative stress” OR “inflammation” OR “cytokine”) AND (“metabolic pathway” OR “glycolysis” OR “OXPHOS” OR “mTOR” OR “AMPK” OR “SIRT1”).

Inclusion criteria encompassed: (1) original research articles (*in vivo*, *in vitro*) and high-quality reviews; (2) studies focusing on the interplay between immunometabolism and male reproductive function; (3) full-text articles available in English or Chinese.

Exclusion criteria were: (1) conference abstracts, editorials, and non-peer-reviewed publications; (2) studies not primarily related to immunometabolic mechanisms; (3) case reports with limited generalizability.

The figure summarizes the literature identification, screening, eligibility, and inclusion steps according to the PRISMA guidelines, culminating in the 90 studies included for qualitative synthesis in this review.

## 3 Fundamental concepts and regulatory mechanisms of immunometabolism

### 3.1 Definition and research background

Immunometabolism is a rapidly evolving discipline at the intersection of immunology and metabolism, centered on the bidirectional regulatory relationship between metabolic pathways and immune cell function (9) immunology emphasizes cytokines, receptor signaling, and transcription factors as the primary regulators of immune cell activation, differentiation, and effector activity. However, recent evidence indicates that the metabolic programs of immune cells are not merely passive reflections of their functional states but active drivers of immune responses and cell fate decisions (10, 11).

#### 3.1.1 Metabolic reprogramming in immune activation

Upon external stimulation, immune cells undergo metabolic reprogramming to rapidly adapt to diverse immune demands. This process not only provides energy and biosynthetic substrates but also influences immune cell fate through metabolic intermediates and signaling pathways (12).

#### 3.1.2 Glycolytic dependency of pro-inflammatory cells

Pro-inflammatory cells (e.g.M1 macrophages, activated Th1/Th17 cells) predominantly rely on enhanced glycolysis to meet their high energy demands and generate metabolic intermediates, such as succinate, which support the synthesis of inflammatory mediators and the production of reactive oxygen species (ROS) (13).

#### 3.1.3 Oxidative metabolism in anti-inflammatory and regulatory cells

In contrast, anti-inflammatory or immunosuppressive cells (e.g.M2 macrophages, Tregs) rely on oxidative phosphorylation (OXPHOS) and fatty acid oxidation (FAO) to maintain long-term energy stability and immune tolerance (14).

This metabolic reprogramming not only reflects but also directs the intensity and direction of immune responses. Under chronic inflammatory or metabolic disorder conditions, such as obesity and diabetes, excessive glycolytic signaling sustains low-grade inflammation, disrupting endocrine balance and reproductive function.

### 3.2 Key metabolic pathways

The regulation of immune cell function depends on the coordinated interaction of multiple energy and substrate metabolic pathways, which together form a complex “metabolic map” underlying immune activation, homeostasis, and disease defense (15).

#### 3.2.1 Glycolysis

Although glycolysis is less energy-efficient, it enables rapid ATP generation under hypoxic or stress conditions, providing essential energy for short-term immune activation (16). In pro-inflammatory cells, upregulated glycolysis and succinate accumulation stabilize HIF-1α, which promotes IL-1β expression and amplifies inflammatory signaling—a central metabolic-inflammatory mechanism (17).

#### 3.2.2 Tricarboxylic acid cycle and oxidative phosphorylation

Under anti-inflammatory or homeostatic conditions, immune cells maintain efficient coupling between the TCA cycle and OXPHOS to sustain cellular energy and support tissue repair (18). M2 macrophages and regulatory T (Treg) cells preserve intact mitochondrial metabolism, enhancing the secretion of IL-10 and other anti-inflammatory mediators to restrain inflammation (19).

#### 3.2.3 Fatty acid metabolism (FAO/FAS)

Fatty acid oxidation (FAO) provides a long-term, sustained energy supply, particularly in anti-inflammatory or memory-type immune cells. In M2 macrophages, FAO supports tissue remodeling, whereas excessive fatty acid synthesis (FAS) promotes membrane remodeling and persistent immune activation (20, 21).

#### 3.2.4 Amino acid metabolism

Glutamine serves as a crucial source of carbon and nitrogen, fueling the TCA cycle and nucleotide biosynthesis. Its metabolite, α-ketoglutarate, acts as a cofactor for epigenetic enzymes, regulating histone and DNA methylation, thereby influencing immune polarization and gene expression (22). Moreover, arginine and tryptophan metabolism modulate immune tolerance and inflammatory balance through substrate depletion and the generation of bioactive metabolites (e.g., kynurenines from tryptophan). By controlling the availability of these critical amino acids, these pathways act as essential ‘metabolic checkpoints’ that can decisively shift immune cell fate towards pro-inflammatory or tolerogenic states, thereby maintaining immune homeostasis (23).

### 3.3 Major regulatory molecules and signaling pathways

Dynamic immunometabolic regulation depends on several energy-sensing and signaling molecules.

#### 3.3.1 mTOR

The mechanistic target of rapamycin (mTOR) is a critical serine/threonine kinase that functions as a central integrator of cellular energy and nutrient status. In immune cells, activation of the mTOR complex 1 (mTORC1) promotes glycolysis, lipid synthesis, and protein translation, thereby driving the activation, proliferation, and effector functions of pro-inflammatory cells such as M1 macrophages and Th17 cells (24, 25). Consequently, mTOR serves as a key molecular link connecting metabolic signaling to inflammatory activation.

#### 3.3.2 AMPK

AMP-activated protein kinase (AMPK) functions as a cellular energy sensor, activated under conditions of low ATP availability. It restores energy homeostasis by promoting fatty acid oxidation (FAO), mitochondrial biogenesis, and autophagy, while simultaneously suppressing anabolic pathways. In the context of immunometabolism, AMPK activation favors the induction of anti-inflammatory phenotypes, such as M2 macrophages and regulatory T cells (Tregs), thereby contributing to the restoration of immune balance (26, 27).

#### 3.3.3 HIF-1α

Stabilized under hypoxic or inflammatory microenvironments, HIF-1α enhances glycolysis and the production of pro-inflammatory cytokines, creating a self-reinforcing metabolic-inflammatory loop (28).

#### 3.3.4 PPARγ and SIRT1

PPARγ, a nuclear receptor, promotes anti-inflammatory phenotypes by activating fatty acid oxidation (FAO) and inhibiting the expression of pro-inflammatory genes (29). SIRT1, an NAD^+^-dependent deacetylase, regulates the acetylation of metabolic enzymes and transcription factors, thereby preserving mitochondrial function and enhancing antioxidant capacity. Together, they act synergistically to maintain immunometabolic balance (30).

## 4 Testicular immune microenvironment and metabolic homeostasis

The testis maintains immune tolerance and metabolic equilibrium through the blood–testis barrier (BTB), an immunosuppressive microenvironment, and the coordinated regulation of multiple cell types (31). This unique immune privilege protects haploid germ cells from autoimmune attack while ensuring efficient spermatogenesis. The maintenance of this privilege is closely linked to metabolic homeostasis, involving the coordinated functions of Sertoli cells, Leydig cells, germ cells, and resident immune cells (32).

### 4.1 Formation of immune privilege

To clarify the terminology used throughout this review, we define the following key concepts:

#### 4.1.1 Testicular immune privilege

This refers to the anatomical and physiological characteristics of the testis that allow it to tolerate auto-antigenic germ cells without eliciting a destructive immune response. It is a property of the organ itself, established by physical barriers (e.g., the blood-testis barrier) and local immunosuppressive mechanisms (33, 34).

#### 4.1.2 Immune tolerance

This describes the functional state of the immune system, specifically its acquired non-reactivity to specific antigens (in this case, sperm antigens) present within the privileged site (35).

#### 4.1.3 Immunosuppressive environment/immune cold environment

These terms are used interchangeably in this text to describe the resultant local microenvironment within the testis, which is rich in anti-inflammatory cytokines (e.g., IL-10, TGF-β) and regulatory immune cells (e.g., M2 macrophages, Tregs) that actively suppress effector immune responses (36, 37).

The establishment and maintenance of testicular immune privilege depend on the dual regulation of structural barriers and immune cell networks. The BTB, composed of tight junctions, adherens, and gap junctions between adjacent Sertoli cells, forms a specialized physical barrier that isolates developing germ cells from systemic immune surveillance (33, 34). Beyond its physical barrier function, local M2 macrophages, regulatory T (Treg) cells, dendritic cells (DCs), and a small number of natural killer (NK) cells secrete anti-inflammatory cytokines such as IL-10 and TGF-β, as well as metabolic regulatory mediators, to cooperatively sustain immune tolerance and local immune equilibrium (35).

### 4.2 Metabolic characteristics and functional differentiation of testicular cells

**Figure 2:**
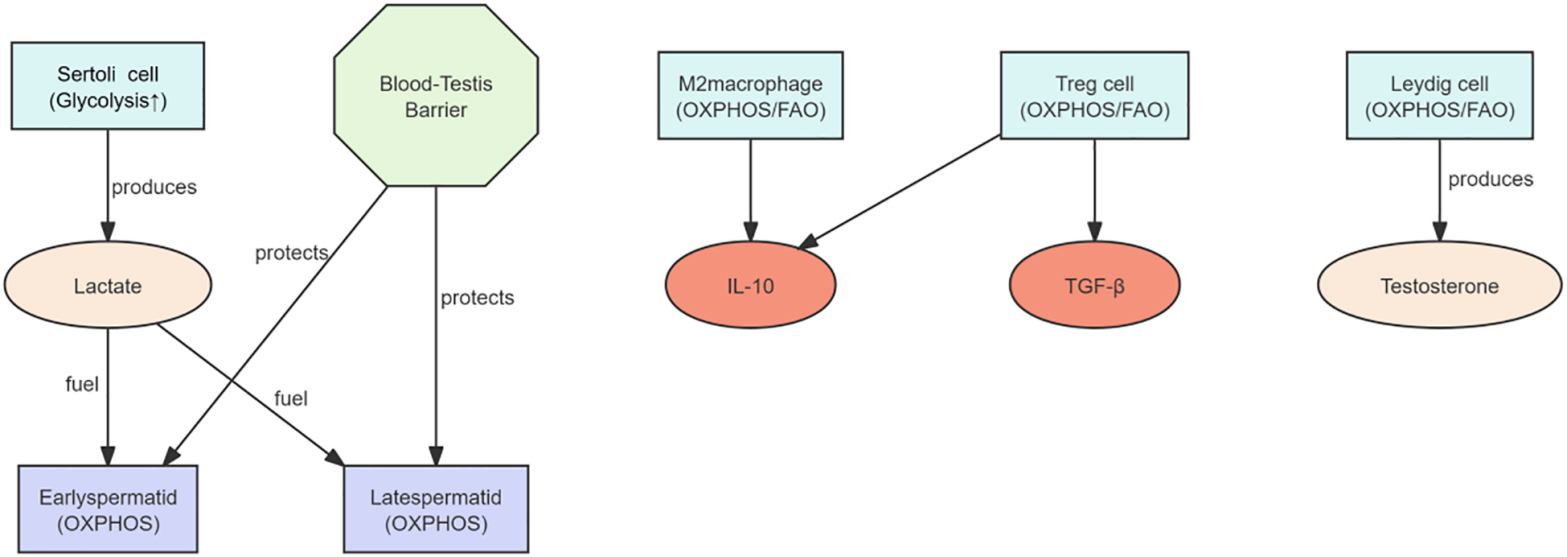
Cooperative interactions between anti-inflammatory immune cells and metabolic processes within the testicular immune microenvironment. In the testicular immune microenvironment, M2 macrophages and regulatory T cells secrete anti-inflammatory cytokines such as IL-10 and TGF-β to maintain local immune tolerance. This anti-inflammatory state is closely linked to specific metabolic patterns, including the high glycolytic activity of Sertoli cells, which produce lactate as an energy substrate for spermatogenesis and support testosterone synthesis in Leydig cells, thereby ensuring normal reproductive function.

Different cell types within the testis exhibit distinct metabolic specializations that directly support their functional differentiation. Sertoli cells rely predominantly on glycolysis, even under normoxic conditions, preferentially metabolizing glucose into lactate to provide energy substrates for developing germ cells (38). Recent studies have shown that lactate acts not only as an energy source for developing germ cells but also as an intra-Sertoli cell signaling molecule that promotes cell survival and regulates oxidative stress responses (39). Leydig cells, in contrast, are metabolically centered on the mitochondrial conversion of cholesterol into testosterone, depending on oxidative phosphorylation (OXPHOS) and fatty acid β-oxidation to sustain energy and substrate supply (40) cells such as macrophages, dendritic cells, and Treg cells maintain anti-inflammatory function and tissue homeostasis through a metabolic pattern characterized by low glycolysis, high fatty acid oxidation, and enhanced OXPHOS (41). This metabolic network collectively maintains the testicular microenvironment in a low-inflammatory and high-antioxidant state, facilitating continuous spermatogenesis and hormone synthesis. As summarized in **Figure 2**.

### 4.3 Interdependence between metabolic homeostasis and immune tolerance

Metabolic homeostasis is fundamental to testicular function and plays a crucial role in mediating immune tolerance. Disturbances in energy metabolism—such as oxidative stress, high-fat diets, and metabolic syndrome—can induce metabolic reprogramming of testicular immune cells, promoting their polarization from the M2 to the M1 phenotype. This repolarization is driven by specific signaling cues; for instance, pro-inflammatory signals like IFN-γ and LPS (via TLR4) promote the M1 state, while anti-inflammatory signals like IL-4 and IL-13 drive the M2 state, with each state being underpinned by distinct metabolic programs (glycolysis for M1, OXPHOS/FAO for M2) (42).

Inflammatory activation suppresses lactate production by Sertoli cells and testosterone synthesis by Leydig cells through multiple mechanisms, disrupting the positive feedback loop between metabolism and immunity. This disruption can contribute to impaired spermatogenesis and hormone deficiency, as observed in models of metabolic syndrome (43). The reciprocal promotion between metabolic imbalance and immune dysregulation has been identified as a central mechanism in the pathogenesis and progression of male infertility and varicocele (44).

Preclinical evidence suggests that strategies aimed at restoring local metabolic homeostasis—such as activating AMPK pathways, promoting fatty acid oxidation (FAO), or employing antioxidant therapies—represent promising avenues worthy of further investigation to improve male reproductive function (45).

## 5 Immune-metabolic imbalance and male infertility

Immune-metabolic imbalance serves as a common molecular basis for various male reproductive disorders. Recent studies indicate that metabolic abnormalities exacerbate inflammatory damage via immune pathways, forming the central pathological axis of “metabolism–immunity–reproduction” (46).

### 5.1 Inflammatory activation and metabolic reprogramming

Infections, environmental toxins, metabolic syndrome, and high-fat diets can induce metabolic reprogramming of immune cells, shifting their metabolism from oxidative phosphorylation-dominant steady-state to glycolysis-dependent pro-inflammatory pathways. This shift exacerbates inflammatory responses and tissue damage by upregulating key signaling pathways, including HIF-1α, mTOR (see Section 3.3.1), and NF-κB. Activation of mTOR, as detailed in Section 3.3.1, promotes a glycolytic metabolic profile in testicular immune cells, sustaining the release of pro-inflammatory cytokines such as IL-6, TNF-α, and IL-1β. This process establishes a chronic low-grade inflammatory state within the testis (47, 48). Excessive accumulation of lactate and reactive oxygen species (ROS) disrupts the blood-testis barrier through multiple signaling pathways, increasing immune cell infiltration and antigen exposure (49). Persistent immune activation not only damages germ cells but may also alter sperm DNA methylation patterns, leading to epigenetic reproductive harm.

### 5.2 Oxidative stress and mitochondrial damage

Oxidative stress serves as a critical link between metabolic disorders and reproductive dysfunction. Collectively, these events outline a core stress pathway in male infertility: metabolic disturbances (e.g., obesity and diabetes) and inflammation increase reactive oxygen species (ROS) generation, which directly damages sperm membranes, mitochondria, and DNA. At the same time, ROS activate stress-sensitive signaling pathways, such as p38 MAPK and NF-κB, which further propagate cellular damage and inflammation, creating a vicious cycle that impairs reproductive function (50, 51). Mitochondrial damage activates caspase-9/3-dependent apoptosis, directly causing germ cell death and structural damage to the seminiferous tubules (52). Moreover, oxidative stress suppresses the expression of steroidogenic enzymes in Leydig cells, further reducing testosterone levels and exacerbating endocrine imbalance (53).

### 5.3 Endocrine–immune–metabolic crosstalk

Male reproductive function depends heavily on precise regulation by the hypothalamic–pituitary–gonadal (HPG) axis, with metabolic hormones and immune signals forming a dynamic, multilayered network essential for reproductive health and systemic homeostasis (54). Insulin and leptin not only regulate energy metabolism but also modulate immune cell activation, differentiation, and function via the PI3K/Akt/mTOR signaling pathway (55). Insulin resistance induces M1 macrophage polarization, promoting chronic low-grade inflammation and the secretion of pro-inflammatory cytokines, thereby establishing a metabolism–immune positive feedback loop (56). Concurrently, inflammatory cytokines (e.g., IL-1β, TNF-α) suppress GnRH and LH secretion, impair Leydig cell responsiveness, and cause hypogonadism (57). Low testosterone levels further inhibit Sertoli cell function and metabolic activity, disrupting the spermatogenic microenvironment and exacerbating male infertility (58). Thus, immunity, metabolism, and endocrine function form a mutually reinforcing pathological loop. Therefore, interventions aimed at breaking this vicious cycle—such as anti-inflammatory nutrition, AMPK activators, or metabolic remodeling drugs—are being investigated as potential strategies for treating male infertility.

## 6 Systemic immune-metabolic dysregulation and reproductive dysfunction

### 6.1 Bridging systemic immunometabolism and testicular dysfunction: convergent mechanisms of action

Systemic metabolic disorders, including obesity, type 2 diabetes, and gut microbiota dysbiosis, do not occur in isolation but collectively contribute to testicular dysfunction through shared immunometabolic pathways (59). This mechanistic connection is often referred to as the “systemic inflammation–metabolic disturbance–reproductive impairment axis,” which centers on the transmission of pro-inflammatory signals and metabolic intermediates from peripheral tissues to the testicular microenvironment (60).

#### 6.1.1 Initiation of systemic inflammatory signaling

**Figure 3:**
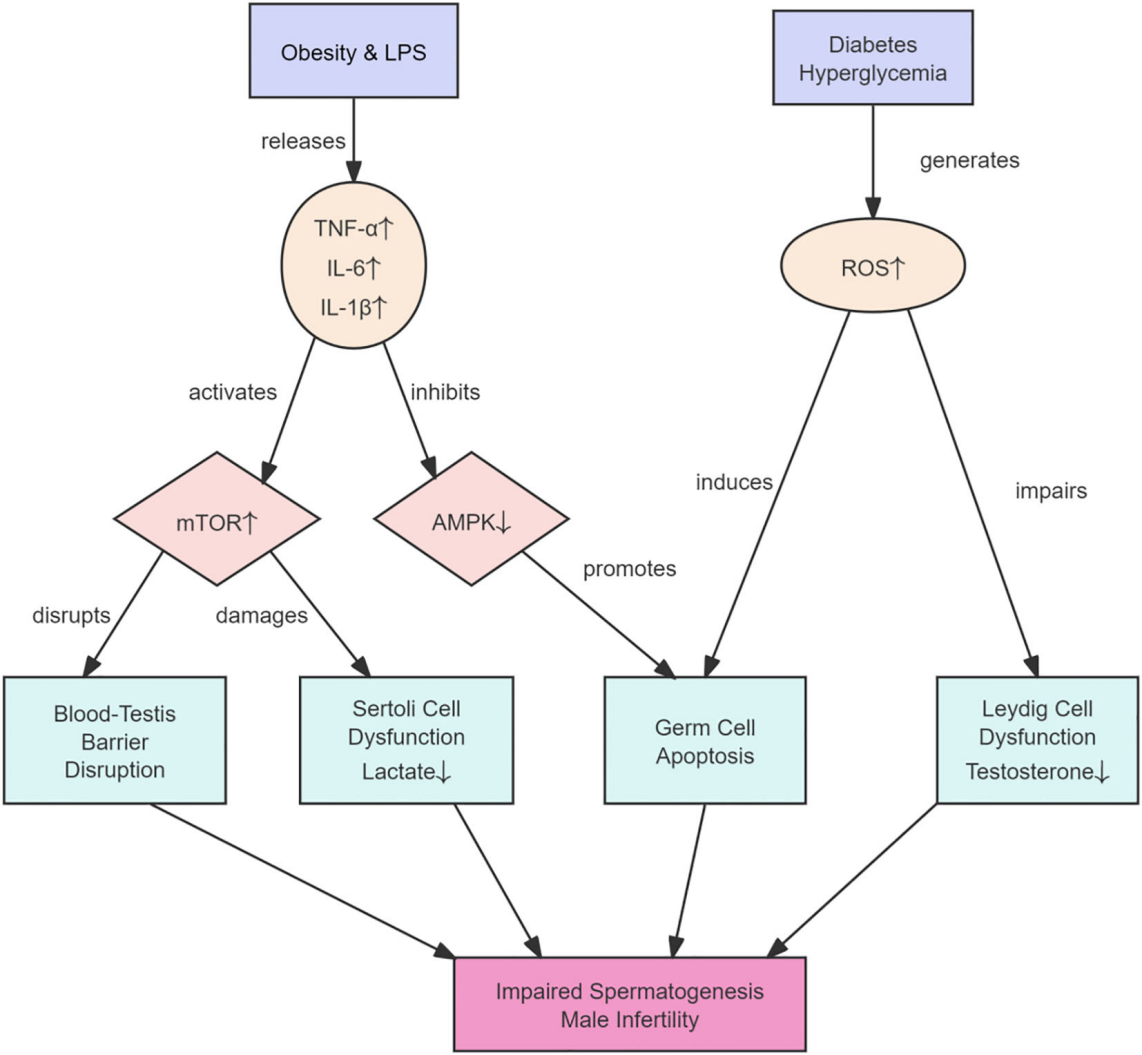
Mechanisms by which systemic metabolic disorders impair male reproductive function through inflammation and oxidative stress. Obesity, diabetes, and other systemic metabolic disorders cause adipose tissue to release pro-inflammatory cytokines such as IL-6 and TNF-α, initiating chronic low-grade systemic inflammation. These cytokines, along with hyperglycemia and lipid accumulation, increase ROS production, which impairs the function of Sertoli and Leydig cells and damages the integrity of the blood-testis barrier. This cascade ultimately reduces sperm quality and androgen synthesis, thereby compromising fertility.

As illustrated in **Figure 3**, this cascade involves the following key steps:

Initiation of Inflammatory Signaling: Obesity-induced adipose tissue inflammation or dysbiosis-driven gut inflammation increases circulating levels of pro-inflammatory cytokines (e.g., TNF-α, IL-6, IL-1β) and bacterial components such as lipopolysaccharide (LPS).

#### 6.1.2 Testicular signal transduction

These systemic factors impair the relative immune privilege of the testis through mechanisms such as compromising the integrity of the blood-testis barrier and activating testicular-resident immune cells (e.g., testicular macrophages) (61).

#### 6.1.3 Intratesticular metabolic reprogramming

Nutrient-sensing pathways activated by hyperglycemia and hyperlipidemia, together with the local inflammatory environment, drive a metabolic shift within testicular cells (62).

Key regulatory components include:

Activation of the mTOR/HIF-1α axis: Inflammatory cytokines and nutrients activate the mTOR and HIF-1α pathways (for their fundamental functions, see Sections 3.3.1 and 3.3.3), promoting a metabolic shift toward glycolysis in both immune and somatic cells, thereby sustaining pro-inflammatory responses.

Suppression of AMPK/SIRT1 activity: The same inflammatory and metabolic stressors often inhibit AMPK and SIRT1 (for their fundamental functions, see Sections 3.3.2 and 3.3.4), reducing their anti-inflammatory, pro-oxidative phosphorylation, and antioxidant effects.

The following sections will detail how specific systemic disorders—obesity (Section 6.2), diabetes (Section 6.3), and gut microbiota dysbiosis (Section 6.4)—converge through this pathway to disrupt male reproductive function.

### 6.2 Obesity impairs spermatogenesis through a multi-faceted immunometabolic cascade

The expansion of adipose tissue, particularly visceral fat, leads to increased infiltration of pro-inflammatory M1 macrophages and the systemic release of cytokines such as TNF-α and IL-6 (63, 64). This chronic, low-grade inflammatory state disrupts the hypothalamic-pituitary-gonadal (HPG) axis and directly affects the testes. Within the testicular microenvironment, these inflammatory signals, combined with nutrient overload (e.g., high glucose and free fatty acids), promote a metabolic shift. This includes aberrant activation of the mTOR and NF-κB pathways, which drive glycolysis and further pro-inflammatory responses in testicular cells (65). Concurrently, energy-sensing pathways such as AMPK are often suppressed, diminishing their anti-inflammatory and antioxidative effects. This metabolic reprogramming disrupts the function of Sertoli cells (compromising the blood-testis barrier) and Leydig cells (reducing testosterone synthesis), and directly induces oxidative stress and apoptosis in germ cells, ultimately leading to reduced sperm count and quality (66).

### 6.3 Diabetes and reproductive function

Diabetes represents a typical state of combined metabolic and immune imbalance. Chronic hyperglycemia induces excessive ROS production, resulting in increased sperm DNA fragmentation and loss of mitochondrial membrane potential (67). Diabetes reprograms T cells and macrophages in the testis, promoting a glycolysis-dependent pro-inflammatory phenotype that secretes interleukin-1β (IL-1β) and interferon-gamma (IFN-γ), thereby exacerbating oxidative stress and apoptosis (68). Moreover, insulin/IGF-1 signaling critically regulates the expression of steroidogenic enzymes in Leydig cells and testosterone production; disruption of this signaling leads to reduced enzyme expression and decreased testosterone secretion (69). Animal studies confirm that diabetic metabolic abnormalities, through the coupling of immune inflammation and energy metabolism disorders, impair testicular mitochondrial function and spermatogonial differentiation, directly affecting spermatogenesis (70).

### 6.4 Gut microbiota–immune–gonadal axis

The gut microbiome has recently emerged as a central hub linking metabolism and reproductive systems. Microbial metabolites, such as short-chain fatty acids and bile acids, regulate immune cell metabolism and function, potentially influencing hormonal regulation and reproductive health (71). A healthy microbiota composition promotes Treg and anti-inflammatory cytokine production, maintaining testicular immune homeostasis (72). Conversely, dysbiosis increases gut permeability, allowing lipopolysaccharides (LPS) and other inflammatory signals to enter the circulation, triggering immune responses and systemic inflammation (73). Clinical observations reveal that obese and diabetic patients exhibit reduced gut microbial diversity, which correlates with decreased serum testosterone levels and abnormal semen parameters. This “gut–immune–gonadal axis” illustrates how peripheral metabolic abnormalities indirectly affect male reproductive function through immune pathways.

## 7 Potential therapeutic targets and interventions

### 7.1 Targeting signaling pathways

**Figure 4:**
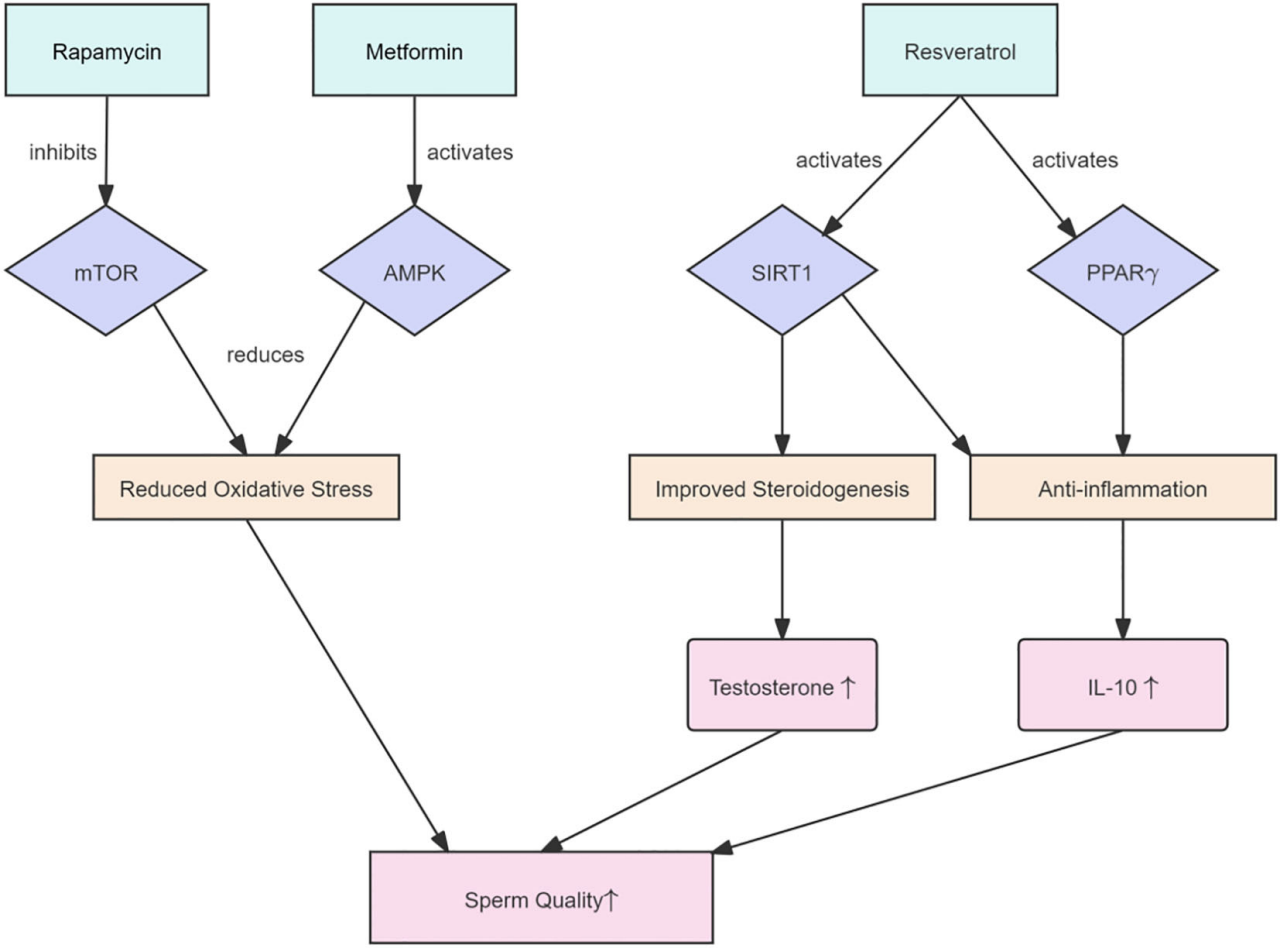
Intervention strategies targeting key immunometabolic signaling pathways to enhance male reproductive function. mTOR, AMPK, and SIRT1 are key regulators of immune-metabolic balance. Rapamycin inhibits overactive mTOR, thereby reducing inflammation; metformin, an AMPK activator, improves glucose and lipid metabolism while decreasing oxidative stress; and resveratrol activates SIRT1, enhancing antioxidant defenses and mitochondrial function. Targeting these pathways presents promising strategies to improve sperm quality and restore hormonal balance.

Several metabolic signaling pathways play a crucial role in maintaining testicular immune homeostasis, as illustrated in **Figure 4**.

#### 7.1.1 mTOR pathway

Given its central role in driving pro-inflammatory metabolic programs, the overactive mTOR pathway observed in conditions of metabolic stress represents a rational therapeutic target (74). Inhibition of mTOR with rapamycin has been shown to reduce testicular oxidative stress and restore anti-inflammatory phenotypes in preclinical models (75). However, it should be noted that, as a potent immunosuppressant, prolonged or high-dose administration of rapamycin may impair spermatogenesis. Multiple studies have demonstrated that rapamycin can induce spermatogenic arrest and disrupt the hypothalamic-pituitary-gonadal (HPG) axis. Therefore, its therapeutic window requires careful evaluation in the context of fertility treatment (76).

#### 7.1.2 AMPK activation as a therapeutic strategy

As a critical energy sensor and promoter of oxidative metabolism (see Section 3.3.2), pharmacological activation of AMPK (e.g., by metformin) can counteract inflammation, enhance the cellular energy supply for spermatogenesis, and support the health of reproductive cells (77). Specifically, in the context of male infertility, AMPK activation is proposed to improve sperm quality through the following mechanisms: (1) reducing the production of pro-inflammatory cytokines in testicular macrophages; (2) alleviating oxidative stress in spermatogenic cells; (3) supporting the integrity of the blood-testis barrier; and (4) improving steroidogenesis in Leydig cells (45).

#### 7.1.3 SIRT1 and PPARγ

These molecules coordinate oxidative stress, mitochondrial homeostasis, and metabolism. Activation of SIRT1 enhances antioxidant defenses and mitochondrial function, while PPARγ primarily regulates lipid metabolism and insulin sensitivity. Their interplay provides a theoretical foundation for therapeutic targeting. Consequently, SIRT1 and PPARγ have emerged as promising therapeutic targets for managing male infertility associated with obesity and metabolic syndrome (78, 79).

### 7.2 Pharmacological and nutritional interventions

This section discusses pharmacological and nutritional interventions aimed at correcting immunometabolic abnormalities, emphasizing their potential to enhance semen parameters, hormonal profiles, and the testicular microenvironment in cases of male infertility. Pharmacological interventions targeting immunometabolic dysfunction have demonstrated improvements in testicular inflammation and sperm parameters in animal models of obesity and diabetes. Metformin, an AMPK activator, has been demonstrated to reduce ROS, improve glucose and lipid metabolism, and support testosterone synthesis (80). However, existing research findings are contradictory; some clinical studies suggest that metformin may impair sperm mitochondrial function, leading to reduced sperm quality in certain male individuals. This underscores the importance of patient stratification and dose optimization in treating male infertility (81). Similarly, rapamycin can suppress mTOR and the release of pro-inflammatory cytokines (82), though its potential adverse effects on spermatogenesis warrant caution (as discussed in Section 7.1.1). Resveratrol, a natural polyphenol, has been shown to activate SIRT1 and exert antioxidant effects and has been shown to ameliorate immunometabolic dysregulation in preclinical studies (83). Furthermore, evidence supports that combining antioxidant supplementation with healthy lifestyle interventions—such as a balanced diet, moderate exercise, and probiotics—can synergistically reduce oxidative stress and systemic inflammation, which are known to be associated with improved sperm DNA integrity in some clinical studies (84).

### 7.3 Emerging research directions

Multi-omics approaches are poised to revolutionize our understanding of testicular immunometabolism. Single-cell RNA sequencing (scRNA-seq) can dissect the heterogeneity of testicular cells and precisely define the metabolic gene expression programs of immune, somatic, and germ cells in healthy and diseased states. Metabolomics can identify and quantify key metabolites (e.g., lactate, succinate, TCA cycle intermediates) within the testis, providing a functional readout of pathway activity and revealing potential diagnostic biomarkers (85, 86). When combined with spatial transcriptomics or proteomics, these techniques can map the precise location of these metabolic and immune states within the tissue architecture, thus revealing how cellular crosstalk within specific niches (e.g., seminiferous tubules) is governed by metabolism (85). Integrating these multi-omics datasets will enable the construction of comprehensive network models of testicular immunometabolism, facilitating the development of precise diagnostic subtyping and personalized intervention strategies for male infertility.

## 8 Future perspectives

Research on immunometabolism provides a novel framework for understanding male reproductive disorders. Future work should transition from association to causation and clinical translation. Addressing these key areas is essential to bridge the gap between current observational knowledge and future clinically actionable insights.

### 8.1 Defining cell-type-specific metabolic vulnerabilities

Future studies must delineate how key pathways like mTOR, AMPK, and SIRT1 are differentially regulated in specific testicular cell types (e.g., Sertoli vs. Leydig vs. tissue-resident macrophages) under various infertile conditions. Utilizing cell-specific knockout models will be crucial to establish causal relationships and identify the most therapeutically relevant cellular targets (87).

### 8.2 High-resolution mapping of the testicular niche

The integration of single-cell and spatial multi-omics technologies (transcriptomics, metabolomics) is needed to create a high-resolution map of the testicular immunometabolic landscape. This will reveal novel cellular interactions, identify dysregulated metabolic checkpoints in patient subpopulations, and uncover biomarkers for infertility subtyping (88).

### 8.3 Translating metabolic reprogramming into therapies

While preclinical studies are promising, the safety, efficacy, and long-term reproductive outcomes of AMPK activators, SIRT1 modulators, and other metabolic drugs require rigorous evaluation in well-designed clinical trials. A critical future direction is to determine whether these interventions can reverse infertility in specific patient subgroups defined by their immunometabolic profile (89).

### 8.4 Exploring the gut–immune–gonadal axis

The role of the gut microbiota and its metabolites (e.g., short-chain fatty acids, bile acids) in regulating testicular immunity and metabolism represents a frontier area. Research should focus on how specific microbial metabolites influence testicular function and whether interventions like probiotics or prebiotics can be developed as adjunct therapies for infertility (90).

## 9 Conclusion

Immune metabolism serves as a central link connecting inflammation, oxidative stress, and male reproductive function. The testis, as an immune-privileged organ, depends on coordinated energy metabolism and immune regulation. When metabolic imbalance and excessive immune activation create a positive feedback loop, oxidative damage, apoptosis, and endocrine disruption collectively impair spermatogenesis and hormone synthesis.

Preclinical evidence indicates that modulating immune–metabolic pathways—inhibiting mTOR, activating AMPK and SIRT1, and promoting fatty acid oxidation—effectively ameliorates inflammation-related and metabolic reproductive disorders in animal models. Integrating multi-omics data to define immune-metabolic subtypes of male infertility is expected to pave the way for more precise diagnostic and therapeutic interventions.

In summary, immune metabolism is a crucial focal point for elucidating the mechanisms of male infertility and a rapidly evolving frontier for fertility-preservation and precision-medicine strategies.
